# A Review of Spatial Analysis Application in Childhood Malnutrition Studies

**DOI:** 10.21315/mjms2022.29.5.4

**Published:** 2022-10-28

**Authors:** Aida Soraya Shamsuddin, Wan Azdie Mohd Abu Bakar, Sharifah Norkhadijah Syed Ismail, Nurul Hazirah Jaafar, Wardah Mohd Yassin, Maisarah Norhizat

**Affiliations:** 1 Department of Nutrition Sciences, Kulliyyah of Allied Health Sciences, International Islamic University Malaysia (IIUM), Pahang, Malaysia; 2 Department of Environmental and Occupational Health, Faculty of Medicine and Health Sciences, Universiti Putra Malaysia (UPM), Selangor, Malaysia

**Keywords:** spatial analysis, Geographic Information System (GIS), mapping, childhood malnutrition

## Abstract

Approximately 230 million children under 5 years old of age suffer from malnutrition and over half of the children below 5 years old deaths are due to malnutrition nowadays. To gain a better understanding of this problem, the application of spatial analysis has risen exponentially in recent years. In this review, the present state of information on the use of spatial analysis in childhood malnutrition studies was evaluated using four databases of digital scientific journals: ScienceDirect, Scopus, PubMed and CINAHL. We chose 2,278 articles from the search results and a total of 27 articles met our criteria for review. The following information was extracted from each article: objective of study, study area, types of malnutrition, subject, data sources, computer software packages, spatial analysis and factors associated with childhood malnutrition. A total of 10 spatial analysis methods were reported in the reviewed articles and the Bayesian geoadditive regression model was the most common method applied in childhood malnutrition studies. This review highlights the importance of the application of spatial analysis in determining the geographic distribution of malnutrition cases, hotspot areas and risk factors correlated with childhood malnutrition. It also provides implications for strategic initiatives to eradicate all forms of malnutrition.

## Introduction

Malnutrition is a complicated issue that is believed to cause more than one-third of all mortality, despite the fact that it is rarely mentioned as the primary cause (1). Undernutrition (wasting, stunting and underweight), micronutrient deficiencies, overweight and obesity are all forms of malnutrition, and children under the age of 5 years old are more vulnerable due to their physical, physiological and cognitive immaturity (2). According to WHO and UNICEF (2, 3), 47 million children under the age of 5 years old were wasted in 2019, 14.3 million were seriously wasted, 144 million were stunted and 38.3 million were overweight or obese. Cases of childhood malnutrition have mainly been reported by low- and middle-income countries. At the same time, child overweight and obesity rates are rising in the same countries (2, 4, 5).

Malnutrition in children is caused by a variety of factors. Poverty has typically been the leading cause of childhood malnutrition (6). Other factors that raise the risk of malnutrition in children include low birth weight, feeding issues, recurrent sickness and chronic disease, and these factors differ by location (7). Climate changes, food insecurity, maternal education, socio-economic status and government policy have also been identified as contributing factors of childhood malnutrition (6). Childhood malnutrition has a wide range of consequences, including an increased risk of illness, mortality and delayed cognitive development, leading to low adult wages and slow economic growth, which can maintain an intergenerational cycle of poverty and illness (1, 4, 7).

Nowadays, the availability of updated satellite imagery and internet data, as well as the usability of geographic information system (GIS) applications, allows for faster innovation and development (8). GIS promotes the interaction of attributes with geographic data to improve the precision of representation and the estimation of spatial analysis. The results from spatial analysis will be more useful than the disorganised data acquired (8). Spatial analysis has been employed in childhood malnutrition research to gain a better understanding of the complexity of childhood malnutrition, which can be influenced by geographical, economic, political and environmental factors (e.g. population density, climatic conditions and diseases) (4). This analysis is based on patterns and underlying processes. It is known as location analysis because it elucidates patterns of personal attributes and geographical presence in terms of geostatistics and geometrics (9). This analysis provides for the spatiotemporal understanding of geographical patterns as well as the identification and analysis of variables related to health behaviours and the resulting outcomes (10). The use of spatial analysis can also be useful in understanding public health issues, such as predicting the population and designing interventions (9, 11).

A review article on the implementation of spatial analysis in childhood malnutrition exploration was carried out by Marx et al. (12). However, this review was geographically limited to childhood malnutrition studies in sub-Saharan Africa (SSA) countries (e.g. Kenya, Mali, Malawi, Sudan, Ethiopia and Somalia). Many studies have been conducted in these countries to address their economic depreciation, which has resulted in a high poverty rate. Therefore, the current review expands on previous work by synthesising the use of spatial analysis in childhood malnutrition studies conducted in other countries of the world, not limited to SSA countries.

The primary objectives of this review were to: i) analyse the use of spatial analysis methods in the studies of the geographic variation of childhood malnutrition and its potential factors; ii) synthesise the spatial analysis methods of the selected studies and separate the methods into three categories: spatial regression model, spatial autocorrelation/clustering and spatial interpolation and iii) evaluate the benefits and disadvantages of using spatial analysis methods in childhood malnutrition studies. The following questions were posed for this review: i) What spatial analysis methods have been used to analyse childhood malnutrition? ii) What spatial analysis method is frequently used in childhood malnutrition studies? iii) What are the benefits and disadvantages of spatial analysis in childhood malnutrition studies? The findings from this review will make a significant contribution to the production of information that can be used to develop policies that improve children’s nutrition security. Furthermore, insights into strategic interventions to eradicate all forms of childhood malnutrition can be obtained. To make the review coherent, we structured it to contain some background information, an objective, a summary of datasets and eligibility, results, discussion and conclusion.

## Methods

### Protocol

The Preferred Reporting Items for Systematic Review and Meta-Analysis Protocols (PRISMA-P) were utilised for this review (13). PRISMA-P is important for some reasons: i) it allows reviewers to carefully prepare and thus predict potential issues; ii) it allows reviewers to record what is anticipated before starting the review and allows others to compare the protocol and the completed review and iii) it prevents arbitrary decision making from occurring (13, 14).

### Information Sources

The systematic search of the present study was conducted on four digital scientific journal databases: ScienceDirect, Scopus, PubMed and CINAHL (EBSCO host interface).

### Selection Process of Articles

#### Identification

The following terms were used to identify related articles in conjunction with keyword searches: ‘spatial’ OR ‘geographic information system’ OR ‘mapping’ AND ‘childhood malnutrition’. The terms were paired with the logical OR operator, while the logical AND operator was used to merge the two terms. Only articles conducting spatial analysis methods to discuss childhood malnutrition were identified as relevant to this review.

### Inclusion and Eligibility Criteria

The articles were chosen for evaluation if they met the requirements for inclusion criteria. The first criterion was the literature type, with the researchers deciding to concentrate solely on journals (research articles) because they served as a primary source of empirical data. As a result, a systematic review, review, meta-analysis, meta-synthesis, book series, books, book chapters and conference proceedings were excluded from this study. This review only included research articles published in English between January 2010 and March 2020. Research articles that have not explicitly reported spatial analysis applications in childhood malnutrition studies were removed from this review.

The titles, abstracts and contents of all articles were fully reviewed to verify that they met the inclusion criteria and were appropriate for use in the current study to meet the objectives. In the first stage, the selection of articles was carried out independently by three reviewers by screening the titles after the database search. The purpose of this stage was to remove duplicate articles. If either of them decided the title of an article was relevant, the article was included—otherwise, the article was excluded. All selected articles were subjected to abstract screening in the second stage. The reviewers must agree to include an article based on the defined relevance criteria during this stage. Any disagreements were resolved by discussion and consensus among all reviewers. When the abstracts of articles could not be presented as relevant or irrelevant, their full-text articles were retrieved for additional review. The full-text articles that met the inclusion criteria were further evaluated and information was retrieved from each article.

### Data Extraction and Analysis

Information from each of the selected articles, such as the objective of the study, study area, types of childhood malnutrition, subject of the study, data sources, computer software packages, spatial analysis (method) and factors associated with childhood malnutrition, was extracted using Microsoft Excel. Then, we further distinguished the spatial analysis methods used in the selected articles into the categories of: i) spatial regression model; ii) spatial autocorrelation/clustering and iii) spatial interpolation. The frequency of each spatial analysis used was tabulated in a table. It should be noted that many types of spatial analysis might be applied in a study.

## Results

### Literature Search Outcomes

**Figure 1:**
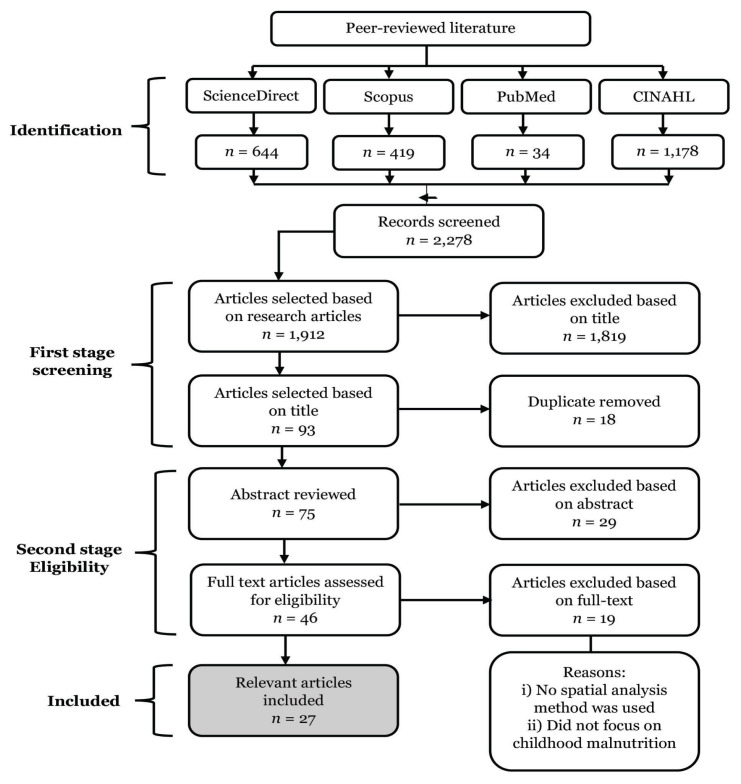
Research flow for the analysis of literature to classify related articles

Our initial search in the ScienceDirect database yielded 644 articles, the Scopus database yielded 419 articles, the PubMed database yielded 34 articles and the CINAHL database yielded 1,178 articles. A total of 2,278 articles were listed, of which 1,912 articles were chosen based on research articles. A total of 1,819 articles were removed based on their title (title screening) and 18 duplicates were removed at the screening stage. In the second stage, 75 abstracts of articles were reviewed and only 46 articles were selected for full-text review. After reviewing the full text for each of the articles, we excluded all articles that did not use the spatial analysis method and did not focus on childhood malnutrition issues (*n* = 19). Thus, a total of 27 relevant articles (1.2% of the total selection) were selected in this review. Figure 1 illustrates the flow of the article’s selection process.

### Description of the Selected Articles

Table 1 lists all the objectives, research areas and years of publication of each selected article. For the objective, most studies (*n* = 21, 77.8%) had a research objective containing geographical terms such as distribution, spatial or geographical variability, spatial clustering, and regional differences. All the studies selected for this review were conducted in developing countries (*n* = 27, 100%). More than half of the studies (*n* = 17, 63%) were conducted in African countries (16, 18, 19, 24, 26, 27, 29–33, 35, 38–41), eight studies were conducted in Asian countries (29.6%) (15, 17, 20–23, 34, 37) and two studies were conducted in American countries (7.4%) (25, 28). Regarding the year of publication, an article was published in 2020 (3.7%) (15), four articles were published in 2019 (14.8%) (16–19) and six articles were published in 2018 (22.2%) (20–25). Next, four articles were published in 2017 (14.8%) (26–29), four articles in 2016 (14.8%) (30–33) and four articles in 2015 (14.85%) (34–37). Apart from these, two articles were published in 2013 (7.4%) (38, 39), one article was published in 2012 (3.7%) (40) and one article was published in 2011 (3.7%) (41).

Table 2 shows further descriptions of the selected articles. Of the 27 articles included in this review, most of the articles (*n* = 21, 77.8%) studied child stunting (15–24, 26–29, 31, 33–35, 37, 38, 41), 13 articles studied childhood wasting (*n* = 13, 48.1%) (15, 16, 18, 20–24, 30, 32, 34, 35, 37), nine articles studied childhood underweight (*n* = 9, 33.3%) (15, 20–22, 24, 30, 34, 37, 39), five studied childhood overweight (*n* = 6, 22.2%) (23, 25, 35, 36, 39, 40) and three studied about childhood obesity (*n* = 3, 11.1%) (25, 39, 40). Most of the selected articles studied more than one type of childhood malnutrition. For subject of the study, 23 articles used children aged under 5 years old as their subject (*n* = 23, 85.2%) (15–18, 20, 21, 23–25, 27–39, 41), one study used children aged under 2 years old (*n* = 1, 3.7%) (19), one study used children aged under 3 years old (0–35 months) (*n* = 1, 3.7%) (26), one study used children aged 3 years old–5 years old (*n* = 1, 3.7%) (40) and one study did not mention the age of children used in the study (*n* = 1, 3.7%) (22).

For information regarding the demographic, socioeconomic status, health and nutrition of the subjects or respondents, most of the articles used data from Demographic and Health Surveys (DHS) (*n* = 15, 55.6%) (16, 19, 21, 23, 24, 26, 30, 31, 33, 35, 36, 38, 39, 40, 41), four studies used data from National Family Health Survey (NFHS) (*n* = 4, 14.8%) (15, 17, 22, 37), two studies obtained data from Food Security and Nutrition Unit (FSNAU) (*n* = 2, 7.4%) (29, 32), two studied from National Nutritional Survey (NNS) (*n* = 2, 7.4%) (20, 34), two studies from Nutritional Status Information System (NSIS) (*n* = 2, 7.4%) (25, 28), one study from Feed the Future Survey (FTF) (*n* = 1, 3.7%) (18), one study from Multiple Indicator Cluster Surveys (MICS) (*n* = 1, 3.7%) (24), one study from Core Welfare Indicators Questionnaire (CWIQ) (*n* = 1, 3.7%) (24) and one study conducted their own survey to obtain the data (*n* = 1, 3.7%) (27).

Climate data (e.g. rainfall, temperature and humidity) was used by six studies (*n* = 6, 22.2%) (17–19, 29, 32, 33), agricultural data (e.g. crop production, enhanced vegetation index (EVI) and normalised difference vegetation index (NDVI)) was used by four studies (*n* = 4, 14.8%) (17, 29, 32, 33), population data (e.g. population density and urbanisation) was used by three studies (*n* = 3, 11.1%) (18, 29, 32), infrastructure data (e.g. irrigation infrastructure and distance to water) was used by two studies (*n* = 2, 7.4%) (18, 32) and topography data (e.g. slope and elevation) was used by two studies (*n* = 2, 7.4%) (19, 35). For software packages for spatial statistics, *R* packages were commonly used (*n* = 9, 33.3%) (15, 16, 20, 21, 23, 27, 29, 32, 36), with four articles using Integrated Laplace Approximation (*R*-INLA) packages (16, 29, 32, 36). The second most commonly used computer software package was ArcGIS (*n* = 8, 29.6%) (17, 19, 22, 28, 30, 31, 39, 40), followed by GeoDa (*n* = 4, 14.8%), WinBUGS (*n* = 3, 11.1%), SaTScan (*n* = 2, 7.4%), BayesX (*n* = 2, 7.4%) and QGIS (*n* = 1, 3.7%). Four articles did not mention the computer software packages they used in their studies (*n* = 4, 14.8%).

### Spatial Analysis Used in Childhood Malnutrition Studies

The spatial analysis used in childhood malnutrition studies by the selected articles is presented in Table 3. The total of different identified spatial analysis methods was 10 methods. These methods were classified into three types of analysis: i) spatial regression model; ii) spatial autocorrelation/clustering and iii) spatial interpolation. Many of these studies applied more than one type of spatial analysis method.

#### Spatial Regression Model

In the spatial regression models used in the included articles, the Bayesian geoadditive regression model was the most used method to investigate the spatial dimension of childhood malnutrition and was applied by eleven studies in this review (*n* = 11, 40.7%) (16, 20, 23, 24, 27, 29, 32, 34, 36, 37, 41). Other than the Bayesian geoadditive regression model, the ordinary least squares (OLS) regression model was also used in four studies (*n* = 4, 14.8%) (17, 18, 22, 33), followed by the spatial error model (SEM) regression model (*n* = 3, 11.1%) (17, 18, 22) and spatial lag model (SLM) regression model (*n* = 2, 7.4%) (17, 22).

#### Spatial Autocorrelation/Clustering

From the selected articles, 23 articles used at least one spatial autocorrelation or clustering method to determine non-random spatial patterns and measure the correlation of spatial observation (Table 3). Global/local Moran’s index was the second most popular method in the articles in this review. It was applied in 10 studies (*n* = 10, 37.0%) (15, 17–19, 22, 25, 26–28, 38). Local indicator of spatial association (LISA) were applied by five studies (*n* = 5, 18.5%) (15, 17, 22, 25, 38). Local Getis-Ord G index (LGi) was also used in three studies (*n* = 3, 11.1%) (31, 39, 40), followed by Kulldorf’s spatial scan analysis (*n* = 3, 11.1%) (26, 30, 35) and semivariogram was used in two studies (*n* = 2, 7.4%) (21, 27).

#### Spatial Interpolation

In this review, only two studies applied the spatial interpolation method in which ordinary kriging was used (*n* = 2, 7.4%) (26, 33). In the study by Barankanira et al. (26), the spatial interpolation method was employed together with a combination of spatial autocorrelation/clustering methods (e.g. Moran’s index and spatial scan analysis). For Lopez-Carr et al. (33), spatial interpolation was applied together with the spatial regression model (e.g. OLS).

## Discussion

In our search of more than 10 years (January 2010–March 2020) of research articles on childhood malnutrition studies, we found that only 27 studies used spatial analysis in their studies. This might be connected to the particular search strategy. We aimed to eliminate false positives, as is the norm in systematic reviews, by choosing search words that were sensitive but not specific. The inclusion of all articles resulted in a vast number of impractical studies, with only a few articles appearing to be relevant. Since we may have overlooked a few articles in this review, we concluded that the specificity of the study did not significantly alter our findings. The small number of chosen articles (*n* = 27) implies that spatial analysis is still new to childhood malnutrition studies. However, considering that spatial analysis is still rarely used in the field of childhood malnutrition, this result is not surprising.

In the selected articles, all studies were conducted in developing countries and more than half of the studies were conducted in the African countries. This finding indicates that factors such as lack of access to food, inadequate health facilities, lack of clean water and sanitation, and lack of child and maternal care in these countries influence the nutritional status of children. The most frequent studies were childhood stunting, followed by childhood wasting and childhood underweight, childhood overweight and childhood obesity. Most of the studies focused on children less than 5 years old. Childhood stunting is the best overall indicator of child development and an accurate depiction of social disparity. Stunting is the most common form of childhood malnutrition, with an estimated 161 million children globally falling below -2 standard deviation (SD) from the median height-for-age value of the WHO Child Growth Standards in 2013 (30, 31).

In public health research, GIS methods are applied in several ways. Spatial data exploration and visualisation are two of the most function of GIS. This review has demonstrated several spatial analysis methods used in childhood malnutrition studies. The Bayesian geoadditive regression model is the most commonly used to investigate the spatial dimension of childhood malnutrition. This method was predominantly used because it promotes the calculation of the conditional association between the state of childhood malnutrition and spatial dependency within the country and across regions (16). The findings obtained provide a deeper explanation of spatial differences in childhood malnutrition patterns of co-existence and aetiology, which would have been ignored in the standard spatial analysis (16). Despite the method’s complexity, the results are acceptable and in line with univariate analysis. This method yields more reliable estimates and a simple understanding of the coefficient of regression described in terms of odds ratios.

Other methods of spatial regression models, such as OLS, SLM and SEM regression, have also been applied by several studies to deepen knowledge of the spatial aspect of childhood malnutrition. The fundamental principle of SLM is that the results of the dependent variables are influenced in the neighbourhood areas, whereas SEM is used to assess the influence of certain variables that are not present in the regression model but have an impact on the output variables. The primary difference between the two models is that, unlike SEM, SLM does not include spatial dependency in the error term. A study by Khan and Mohanty (22) found that SEM was better functioning and thus provided a final assessment of the relationship in their study.

The spatial autocorrelation/clustering is a method for determining the existence and absence of non-random spatial patterns in a study area. These approaches, also known as hotspot analysis, yield estimates disaggregated to the spatial unit level to characterise significantly autocorrelated local areas. In this review, this method was used by the selected articles to identify areas where childhood malnutrition cases are higher or lower than expected under the assumption that childhood malnutrition cases are uniformly distributed spatially. If the cases are not random, this method helps identify significant spatial clusters. Applying this analysis to childhood malnutrition studies can facilitate improved regional targeting by stratifying areas of higher risks and identifying physical locations that are important for the design of nutrition intervention programmes in target regions (39). Targeted regional needs for interventions can have the utmost benefit in meeting the needs of the population relative to random intervention (21). The use of this method also has limitations, particularly in the selection of significant clusters. In spatial analysis, there is no specific cluster type that clearly suits all data—they are all dependent on the purpose of the study as well as on the quality of the data input. Ignoring certain risks of covariate factors may affect the factors responsible for the observed clustering of malnutrition. This is due to the possibility that the non-random distribution of risk factors in the study area may have a role in the clustering process (35). The circular cluster type was employed by the majority of the researchers to identify and physically locate significant clusters of childhood malnutrition. The circular cluster may not accurately represent the real scenario and types of clusters, such as elliptical, irregularly shaped, long and narrow clusters, may be more significant to the model. This constraint has practical implications, particularly in geographically targeted interventions, because the selection of respondents within a circular cluster may or may not represent the study community.

Spatial interpolation is a method of estimating values at an unsampled location using a network of known-valued points (11). The kriging, inverse distance weighting (IDW), spline and polynomial interpolation are the most common spatial interpolation methods (11). In the selected articles, only the ordinary kriging method was used in two studies. Although spatial interpolation provides several methods, there is no ‘rule of thumb’ for which method is best in which circumstances. Even when utilising the same method and data input point, different parameters might produce different surfaces. As a result, it is critical to assess and comprehend the accuracy and reliability of surface data generated through spatial interpolation. This method should not be based solely on quantitative assessment. The use of 2D and 3D visualisations can assist in revealing information that is not detectable through quantitative assessment. However, without complementing quantitative assessment, the use of spatial analysis methods cannot be achieved. This is because the spatial components of data in a GIS are fundamentally quantitative, as they consist of coordinate pairs—numbers that can be statistically manipulated. Spatial analysis uses this either to summarise the patterns within spatial data or to give details on how attribute data are arranged in space.

The selected articles showed that many factors contribute to childhood malnutrition. The determinants of the nutritional condition of children can be classified into several factors, such as: i) proximate factors e.g. age of child, sex of child, birth size, insufficient food intake and infection; ii) distal factors e.g. sociocultural, economic, environmental and climatic variables; and iii) intermediate factors e.g. wealth index, mothers’ ethnicity and level of education of mother and father. Depending on geographical and climatic conditions and anthropogenic influences, these factors are believed to differ temporally and spatially (29). Therefore, the assessment of childhood malnutrition must consider such variations. The applications of spatial analysis in childhood malnutrition studies provided by the selected articles undeniably offer a captivating resource for a deeper understanding of the fine geographical differences and essential causes of childhood malnutrition (20). The geographic location is an essential variable of recognised malnutrition predictors and is linked to food security and accessibility.

The potential drawbacks of this review are the omission of non-English articles and limited search terms, which could have contributed to the unintended exclusion of studies. This review was dominated by cross-sectional studies. This suggests that the study focused on data collected at a particular point in time, making it difficult to discover patterns in childhood malnutrition status over time. The cross-sectional studies are efficient, inexpensive and simple to conduct; however, they may cause the researcher to be biased in their depiction of the result, which may impact secondary data (42). A longitudinal study, by contrast, will reveal innovations, patterns and improvements across time (42). As a result, the findings of this study do not provide a comprehensive assessment of the factors influencing children’s nutritional status.

## Conclusion

Many studies have conducted various spatial analysis methods by combining national and regional childhood malnutrition data and attempts have been made to illustrate the geographic distribution of childhood malnutrition using a map. The methodological strength of such studies spans from mapping and advance statistical spatial analyses through the identification of environmental and other geographical factors on childhood malnutrition. As a result, researchers, policymakers and programme managers have acknowledged location as a crucial determinant of population and health outcomes. This is especially true in the case of childhood malnutrition, which can be impacted by geographical, cultural and environmental factors. According to the findings of this review, the application of spatial analysis methods in childhood malnutrition research is important for understanding the geographic distribution of malnutrition cases, hotspot areas and risk factors associated with childhood malnutrition. To assist the government, policymakers and other authorities in implementing strategies and designing intervention programmes in high-risk areas to reduce malnutrition in children throughout the world, it is necessary to evaluate and understand the geographical distribution of childhood malnutrition cases.

## Figures and Tables

**Table 1:** Objectives of the reviewed articles (*n* = 27)

Author	Year	Research area	Objective
Liou et al. (15)	2020	India	Identify child malnutrition distribution and the inequality of income within and across districts
Adeyemi et al. (16)	2019	Burkina Faso and Mozambique, sub-Saharan Africa	Investigate disparities in small scale geographical variability and the potential risk factors for children health outcomes (anaemia, stunting and wasting)
Bharti et al. (17)	2019	640 districts of India	Examine the geographical distribution of childhood stunting and explore factors that cause childhood stunting
Cooper et al. (18)	2019	Ghana and Bangladesh	Examine the impact of precipitation extremes on household food security and child undernutrition
Uwiringiyimana et al. (19)	2019	Rwanda	Investigate the social, socio-economic, and environmental variables that influence the spatial patterns of stunting
Akseer et al. (20)	2018	Afghanistan	Analyse district-level geographical differences and nutritional status factors (stunting, wasting or underweight) between women and children
Hasan et al. (21)	2018	Bangladesh	Assess changes in the spatial clustering of child malnutrition below 5 years old of age
Khan and Mohanty (22)	2018	India	Evaluate the relationship between spatial variability and child malnutrition
Mia et al. (23)	2018	Bangladesh	Investigate socio-demographic and regional differences in the prevalence of under and over nutrition by children and mothers
Osgood-Zimmerman et al. (24)	2018	51 African countries	Examine the child growth failure by using geospatial analysis
Torres-Roman et al. (25)	2018	Peru	Study the prevalence of overweight and obesity in children
Barankanira et al. (26)	2017	Côte d’Ivoire, West Africa	Analyse variations childhood stunting over time, describe spatial distribution and identify spatial clusters of childhood stunting
Hagos et al. (27)	2017	Ethiopia	Identify risk factors for childhood stunting and examine the impact of spatial reliance on risk factors detection of stunting
Hernández-Vásquez and Tapia-López (28)	2017	Peru	Evaluate variations in geographic prevalence of childhood malnutrition
Kinyoki et al. (29)	2017	Somalia	Quantify spatial co-occurrence of acute respiratory infections, diarrhoea and childhood stunting
Alemu et al. (30)	2016	Ethiopia	Identify the spatial clusters of child undernutrition
Haile et al. (31)	2016	Ethiopia	Determine spatial variability and factors related to child stunted growth
Kinyoki et al. (32)	2016	Somalia	Determine the sub-national seasonal prevalence and patterns in childhood wasting
López-Carr et al. (33)	2016	Lake Victoria Basin, Burundi, Kenya, Rwanda, Tanzania, and Uganda	Create a spatially and temporally coherent dataset of the components of climate exposure linked to undernutrition susceptibility in children
Cesare et al. (34)	2015	143 districts in Pakistan	Evaluate the relationship between nutritional status with food security, maternal and household socioeconomic factors
Gebreyesus et al. (35)	2015	Meskane Mareko districts, Ethiopia	Investigate the local spatial structure of stunting and wasting among children
Mtambo et al. (36)	2015	Malawi	Explain variability in childhood overweight by fitting a modern spatial quantile model
Yadav et al. (37)	2015	India	Investigate the influence of geographical context on child malnutrition
Adekanmbi et al. (38)	2013	37 states in Nigeria	Examine and describe the variation between states in childhood stunting
Turi et al. (39)	2013	Uganda	Identify risk factors for underweight, overweight, and obesity in mothers and children and determine the hotspot areas
Pawloski et al. (40)	2012	Rural and urban areas of Kenya	Assess the relationship between spatial variations with mothers and children
Kandala et al. (41)	2011	Democratic Republic of Congo	Investigate the effect of spatial heterogeneity on child nutritional status

**Table 2:** Description of the reviewed articles (*n* = 27)

Author	Types of malnutrition	Children age	Data	Computer software packages	Spatial analysis (method)	Factors associated with child malnutrition
Liou et al. (15)	Stunting, wasting and underweight	< 5 years old	- Demographic, health and nutrition (NFHS)	R	- Moran’s Index - Local Indicators of Spatial Association (LISA)	- Household wealth
Adeyemi et al. (16)	Stunting and wasting	< 5 years old	- Demographic, health and nutrition (DHS)	R-INLA WinBUGS	- Bayesian geoadditive regression model	- Household poverty - Morbidity - Short birth interval - Breastfeeding practices - Antenatal attendance - Maternal literacy
Bharti et al. (17)	Stunting	< 5 years old	- Demographic, health and nutrition (NFHS) - Socio-economics - Rainfall - Temperature - Crop production	ArcGIS GeoDa	- Moran’s Index - LISA - Ordinary least squares (OLS) - Spatial lag model (SLM) - Spatial error model (SEM)	- Temperature - Rainfall - Socio-economic status - Crop production
Cooper et al. (18)	Stunting and wasting	< 5 years old	- Demographic, health and nutrition, food security (FTF) - Rainfall - Population density - Irrigation infrastructure	NA	- Moran’s Index - OLS - SEM	- Drought - Rainfall
Uwiringiyimana et al. (19)	Stunting	< 2 years old	- Demographic, health and nutrition (DHS) - Elevation - Slope - Temperature - Rainfall - Relative humidity	ArcGIS	- Moran’s Index	- Child’s age - Child’s sex - Mother’s height - Maternal education - Birth weight - Elevation - Being served by a rural market
Akseer et al. (20)	Stunting, wasting and underweight	< 5 years old	- Demographic, health and nutrition (NNS)	WinBUGS R	- Bayesian geoadditive regression model	- Household wealth - Maternal literacy - Maternal - anthropometry - Child’s age - Food security - Geographical location - Infrastructure
Hasan et al. (21)	Stunting, wasting and underweight	< 5 years old	- Demographic, health and nutrition (DHS)	R	- Semivariogram analysis	- Geographical location - Socio-economic status - Poverty
Khan and Mohanty (22)	Stunting, wasting and underweight	NA	- Demographic, health and nutrition (NFHS)	ArcGIS GeoDa	- Moran’s Index - LISA - OLS - SLM - SEM	- Poverty - Mother’s body mass index - Maternal education - Infrastructure - Breastfeeding practices
Mia et al. (23)	Stunting, wasting and underweight	< 5 years old	- Demographic, health and nutrition (DHS)	R	- Bayesian logistic regression model	- Maternal education - Poverty - Geographical location
Osgood-Zimmerman et al. (24)	Stunting, wasting and underweight	< 5 years old	- Demographic, health and nutrition (DHS, MICS, CWIQ)	NA	- Bayesian geoadditive regression model	- Socio-economic status - Born early - Born small
Torres-Roman et al. (25)	Overweight and obesity	< 5 years old	- Demographic, health and nutrition (NSIS)	GeoDa	- Moran’s Index - LISA	- Geographical location
Barankanira et al. (26)	Stunting	0 month old–35 months old	- Demographic, health and nutrition (DHS)	QGIS	- Ordinary kriging - Moran’s Index - Spatial scan analysis	- Socio-economic status
Hagos et al. (27)	Stunting	< 5 years old	- Demographic, health and nutrition - Food insecurity (HFIAS) - Socioeconomic	R WinBUGS	- Semivariogram analysis - Moran’s Index - Bayesian geoadditive regression model	- Child’s age - Child’s sex - Maternal education - Food security
Hernández-Vásquez and Tapia-López (28)	Stunting	< 5 years old	- Demographic, health, nutrition (NSIS)	ArcGIS GeoDa	- Moran’s Index	- Urbanization
Kinyoki et al. (29)	Stunting	< 5 years old	- Demographic, health and nutrition (FSNAU) - Rainfall - Enhanced vegetation index (EVI) - Temperature - Urbanisation	R-INLA	- Bayesian geoadditive regression model	- Rainfall/drought - Vegetation cover - Access to foods in high protein and carbohydrates - Child’s age - Child’s sex - Illness - Temperature
Alemu et al. (30)	Wasting and underweight	< 5 years old	- Demographic, health and nutrition (DHS)	ArcGIS SaTScan	- Spatial scan analysis	- Geographical location
Haile et al. (31)	Stunting	< 5 years old	- Demographic, health and nutrition (DHS)	ArcGIS	- Local Getis-Ord G index (LGi)	- Short birth interval - Child’s sex - Household head’s sex - Severe anaemia - Maternal education - Father’s education - Mother’s body mass index - Socio-economic status - infrastructure
Kinyoki et al. (32)	Wasting	< 5 years old	- Demographic, health and nutrition (FSNAU) - Rainfall - EVI - Temperature - Distance to water - Urbanisation	R-INLA	- Bayesian geoadditive regression model	- Precipitation - EVI - Temperature
López-Carr et al. (33)	Stunting	< 5 years old	- Demographic, health and nutrition (DHS) - Rainfall - Normalised difference vegetation index (NDVI)	NA	- Ordinary Kriging - OLS	- Rainfall - NDVI
Cesare et al. (34)	Stunting, wasting, underweight and overweight	< 5 years old	- Demographic, health and nutrition (NNS) - Socioeconomic - Food security	NA	- Bayesian geoadditive regression model	- Mother’s height - Mother’s weight - Poverty - Maternal education - Food security
Gebreyesus et al. (35)	Stunting and wasting	< 5 years old	- Demographic, health and nutrition (DHS) - Food security - Elevations	SaTScan	- Spatial scan analysis	- Elevation of the houses - Place of residence - Household dietary diversity - Food security - Infrastructure
Mtambo et al. (36)	Overweight	< 5 years old	- Demographic, health and nutrition (DHS)	R-INLA	- Bayesian geoadditive regression model	- Child’s sex - Household head’s sex - Type of residence - Mother’s working status - Vitamin A
Yadav et al. (37)	Stunting, wasting and underweight	< 5 years old	- Demographic, health and nutrition (NFHS)	BayesX	- Bayesian geoadditive regression model	- Illness - Child’s sex - Birth order - Vitamin A
Adekanmbi et al. (38)	Stunting	< 5 years old	- Demographic, health and nutrition (DHS)	GeoDa	- Moran’s index - LISA	- Poverty - Higher health deprivation index
Turi et al. (39)	Underweight, overweight and obesity	< 5 years old	- Demographic, health and nutrition (DHS)	ArcGIS	- LGi	- Geographical location
Pawloski et al. (40)	Overweight and obesity	3 years old–5 years old	- Demographic, health and nutrition (DHS)	ArcGIS	- LGi	- Urbanisation - Cultural differences - Geographical location
Kandala et al. (41)	Stunting	< 5 years old	- Demographic, health and nutrition (DHS)	BayesX	- Bayesian geoadditive regression model	- Child’s sex - Geographical location - Maternal education - Socio-economic status

**Table 3:** Classification of spatial analysis methods of the reviewed articles (*n* = 27)

Spatial analysis methods	Number of articles	Percentage	Reference
Spatial regression model			
Bayesian geoadditive regression model	11	40.7%	16, 20, 23, 24, 27, 29, 32, 34, 36, 37, 41
Ordinary least squares (OLS) regression model	4	14.8%	17, 18, 22, 33
Spatial error model (SEM) regression model	3	11.1%	17, 18, 22
Spatial lag model (SLM) regression model	2	7.4%	17, 22
Spatial autocorrelation/clustering			
Global/Local Moran’s index	10	37.0%	15, 17–19, 22, 25, 25–28, 38
Local indicators of spatial association (LISA)	5	18.5%	15, 17, 22, 25, 38
Local Getis-Ord G Index (LGi)	3	11.1%	31, 39, 40
Kulldorf’s Spatial scan analysis	3	11.1%	26, 30, 35
Semivariogram	2	7.4%	21, 27
Spatial interpolation			
Ordinary Kriging	2	7.4%	26, 33
